# Letter to the Editor

**DOI:** 10.1002/pds.3283

**Published:** 2012-04-19

**Authors:** Nicolas Deltour, Maylis Coste, Isabelle Tupinon-Mathieu

**Affiliations:** 1 Department of Biostatistics and Pharmacoepidemiology, Institut de Recherches Internationales ServierSuresnes, France; 2 Institut de Recherches Internationales ServierSuresnes, France

To the Editor,

In the recent article by Fournier and Zureik^1^ “Estimate of deaths due to valvular insufficiency attributable to the use of benfluorex in France,” the authors concluded that “benfluorex is likely to be responsible for around 3100 hospitalizations and 1300 deaths due to valvular insufficiency,” and that “these figures may be underestimated.” This analysis constitutes the third published extrapolation of mortality with benfluorex.^2^–^3^ The latest estimate is based on the product of the number of hospitalizations, etiologic fractions, and a ratio of consumption, using the same original data^4^–^6^ as in calculations by Hill,^2^–^3^ completed with new hypotheses and data sources on benfluorex consumption (unpublished data) and rate of mortality.^7^

We have five major concerns regarding the methodology of these projections, all of which affect the conclusions that can be drawn by Fournier and Zureik and with which we disagree.

1. The estimations are based on the only available data on benfluorex-associated risk of hospitalization for valvular disease in diabetics, that is, a study by Weill *et al*.^4^ performed in French medico-administrative database with fundamental biases. These very large administrative databases contain very limited information on risk factors (e.g., smoking or body mass index are not recorded) and comorbidities (e.g., only severe and costly long-term diseases are included, i.e., “*affection longue durée*,” [ALD]). The methodology of Weill *et al*. fails to address selection and classification biases in the generation of both cohorts: benfluorex-exposed (43 044 patients) and non-exposed (1 005 129 patients). Patients exposed to benfluorex are treated in combination with at least one other antidiabetic treatment. Thus, the absolute risk level of valvular insufficiency could be higher in the benfluorex cohort. A matching method on major risk factors should have been performed (e.g., based on a propensity score). Even if the statistical model is adjusted for sex, age, and ALD, it cannot be considered as being able to overcome the intrinsic differences between the two cohorts. Moreover, Weill *et al*. appear to consider ALD as a composite criterion, defined as at least one of coronary artery disease, peripheral artery disease, heart failure, and cerebrovascular disease. This is an incomplete adjustment because it does not consider the effect of each pathology separately, or interactions between diseases or with benfluorex. In this way, the estimation of relative risk (RR) of 3.1 (95% CI 2.4–4.0) is questionable and could be an overestimation of the increased risk due to benfluorex.
2. Although it may be clinically relevant to define a minimal level of benfluorex exposure above which there is an excess of risk, the rationale behind the 30-box threshold set by Fournier and Zureik is also questionable. This value is based on the study by Weill *et al*.^5^–^6^ on a cohort of 303 336 patients exposed to benfluorex followed from 2006 to 2009. The threshold of 30 boxes is based on hypothetical previous exposure in the fraction of the cohort who were considered to have been exposed to benfluorex prior to 2006 and then extrapolated to the whole cohort. This hypothesis leads authors to consider that the 597 observed hospitalizations in the 303 336 patients (who had consumed 10 million boxes) were all attributable to the use of benfluorex. It would have been more reliable to base the threshold on a time-to-event approach according to treatment exposure in the de novo benfluorex patients. Furthermore, on the basis of 1075 patients in the E3N cohort (unpublished data), Fournier and Zureik considered that 78 million boxes of benfluorex (out of 145 million, i.e., total sales over three decades) had been prescribed to patients already exposed to at least 30 boxes. Finally, Fournier and Zureik go on to apply the simple multiplicative coefficient of 78 million boxes/10 million boxes in the projection, producing the figure of 4531 hospitalizations, via a whole string of complex and questionable assumptions.
3. Fournier and Zureik used a maximalist rate of mortality (43% [95% CI 38%–47%], because of an RR = 1.75 [95% CI 1.61–1.90]) from the North American general population^7^ based on 971 patients compared with a theoretical matched sample. This rate of death is based on moderate or severe valvular heart diseases of varied etiology. However, in the same report,^7^ there was a second mortality rate (26% [95% CI 13%–38%] based on an adjusted RR = 1.36 [95% CI 1.15–1.62]) estimated from a pooled population-based studies (12 000 patients), which appeared more reliable because it included severe, moderate, and also less severe valvular diseases,^1^ which are closer to the valvular diseases observed in the general population as well as those observed with benfluorex. It should be noted that the benfluorex-associated increase in risk for onset of valvular insufficiency has been reported to almost disappear 2 or 3 years after treatment is stopped.^1^, ^5^–^6^ Moreover, the RR = 1.36 was a formal comparison between two cohorts (with and without valvular disease) and was adjusted on major risk factors. Finally, we question the pertinence of transposing North American results to a French population; indeed, it is noteworthy that the rate of death available for the 303 336 patients^5^–^6^, ^8^–^9^ is much lower (46 valvulopathy-attributable deaths out of 597 hospitalizations, leading to a rate of 7.7%). The omission of this rate in Fournier and Zureik's calculations is questionable. According to these choices, the corresponding etiologic fraction decreases dramatically from 43% to 26%, or even to 7.7%.
4. Fournier and Zureik hypothesized that their estimations in a diabetic population aged 40 to 69 years between 2006 and 2009^4^ could be generalized to all users of benfluorex over three decades. This hypothesis is fundamental to a reliable estimate,^10^ but is highly questionable. Would the RR estimated in diabetic patients (3.1 [95% CI 2.4–4.0]) be the same, whatever the treated population and without consideration of potential interaction with benfluorex? Has the absolute risk of valvular heart disease been the same in the general population over the last 30 years? In other words, according to the authors, disease profiles and clinical practice have not changed for three decades.^10^
5. Finally, the estimates of authors are based on numerous interwoven hypotheses. Beyond the understanding of the rationale of these hypotheses, their links with each other and their applications, we maintain that the greater the number of hypotheses, the greater the uncertainty.^10^ The authors do make some attempt to address uncertainty, but this was not applied systematically.

In this context, it is striking that Fournier and Zureik start from the same dataset as Hill, make a different series of hypotheses and assumptions, to arrive at a three times higher number of events with benfluorex. This demonstrates the impact of making successive and questionable assumptions on the reliability of such estimations. With this in mind, we disagree with the conclusions of the authors on the estimate of deaths.
